# Activation of NF-κB driven inflammatory programs in mesenchymal elements attenuates hematopoiesis in low-risk myelodysplastic syndromes

**DOI:** 10.1038/s41375-018-0267-x

**Published:** 2018-10-12

**Authors:** Zhen Ping, Si Chen, Sjoerd J. F. Hermans, Keane J. G. Kenswil, Jacqueline Feyen, Claire van Dijk, Eric M. J. Bindels, Athina M. Mylona, Niken M. Adisty, Remco M. Hoogenboezem, Mathijs A. Sanders, Eline M. P. Cremers, Dicky J. Lindenbergh-Kortleve, Janneke N. Samsom, Arjan A. van de Loosdrecht, Marc H. G. P. Raaijmakers

**Affiliations:** 1 Department of Hematology, Erasmus MC Cancer Institute, Rotterdam, The Netherlands; 2 Department of Hematology, VU University Medical Center, Amsterdam, The Netherlands; 3 Laboratory of Pediatrics, Division of Gastroenterology and Nutrition, Erasmus University Medical Center, Rotterdam, The Netherlands

**Keywords:** Myelodysplastic syndrome, Haematopoietic stem cells, Cancer microenvironment

## Abstract

- Activation of NF-κB signaling in mesenchymal cells is common in LR-MDS.
- Activation of NF-κB in mesenchymal cells leads to transcriptional overexpression of inflammatory factors including negative regulators of hematopoiesis.
- Activation of NF-κB attenuates HSPC numbers and function ex vivo.

Myelodysplastic syndromes (MDS) are clonal disorders characterized by ineffective hematopoiesis and the propensity for leukemic transformation. Cumulating evidence has challenged the traditional view that MDS is exclusively driven by hematopoietic cell intrinsic factors. Mesenchymal cells in the bone marrow (BM) microenvironment have emerged as key players in disease pathogenesis, as either initiating or contributing factors [1–4]. We have earlier demonstrated that the transcriptional landscape of highly purified mesenchymal elements from human low-risk MDS (LR-MDS) is distinct from normal mesenchymal cells and characterized by cellular stress and the upregulation of inflammatory molecules with known inhibitory effects on normal hematopoiesis [4]. Specifically, mesenchymal overexpression of the alarmins S100A8/9 was shown to drive genotoxic stress in hematopoietic stem/progenitor cells (HSPCs) and is related to leukemic evolution in a subset of LR-MDS patients [3]. An important question emerging from these findings is the nature of the upstream drivers of cellular stress and inflammatory programs in LR-MDS mesenchyme. Here, we show that activation of NF-κB in mesenchymal cells is common in LR-MDS, driving transcriptional activation of inflammatory programs and attenuating HSPC function.

We earlier reported on the elucidation of the transcriptome of highly purified mesenchymal cells isolated from LR-MDS patients (*n* = 45) by massive parallel RNA sequencing [3], suggesting inflammation in these mesenchymal elements. In order to identify candidate master regulatory pathways upstream of inflammatory programs in LR-MDS, we performed Gene Set Enrichment Analysis (GSEA) comparing the transcriptomes of these 45 patients to mesenchymal cells purified from healthy controls (*n* = 10) [4]. In total, 120 gene signatures were significantly enriched in the LR-MDS mesenchyme, while 8 signatures were enriched in normal mesenchymal cells. Among the signatures upregulated in LR-MDS patients was a remarkable abundance of signatures related to the activation of the nuclear factor-kappa B (NF-κB) family of transcription factors (Fig. 1a, b). To corroborate the notion of activation of this pathway and provide better insight into the heterogeneity within the population, we assessed the expression levels of NF-κB inhibitor *NFKBIA* (also known as *IκB-ɑ*), which forms an autoregulatory loop with activated NF-κB transcription factors and therefore directly reflects activation of NF-κB signaling [5, 6]. Overexpression of *NFKBIA* was found in the majority of patients, suggesting that mesenchymal NF-κB activation is a common feature in LR-MDS (Fig. 1c). To confirm the functional activation of NF-κB in mesenchymal elements in LR-MDS, we demonstrated increased phosphorylation of p65, a component of the activated NF-κB complex, in intramedullary located CD271^+^ mesenchymal cells (Fig. 1d) as well as in bone-lining CD271^+^ stromal cells (Fig. 1e, f). Moreover, pathway analysis (GSEA) confirmed the transcriptional activation of NF-κB signaling in patients with increased *NFKBIA* expression in their mesenchymal niche cells (Figure S1A and S1B). *NFKBIA* expression was significantly correlated with the expression of inflammatory cytokines and negative regulators of hematopoiesis, which are bona fide NF-κB downstream targets such as *IL6*, *IL8*, and *CCL3* (Figure S1C). No correlation was found between *NFKBIA* expression and expression of *S100A8* or *S100A9* (spearman correlation −0.11 and −0.22; *P*-adjusted 0.62 and 0.28, respectively). Patients with activated NF-κB signaling (*NFKBIA*^+^) in mesenchymal niche cells had no significant difference in overall or progression-free survival in comparison to the *NFKBIA*^−^ subset (Figure S2A and S2B) in this cohort of uniformly treated LR-MDS patients [4]. No significant correlations were found between mutational status and activation of NF-κB signaling in mesenchymal cells (Figure S2C).

**Fig. 1:**
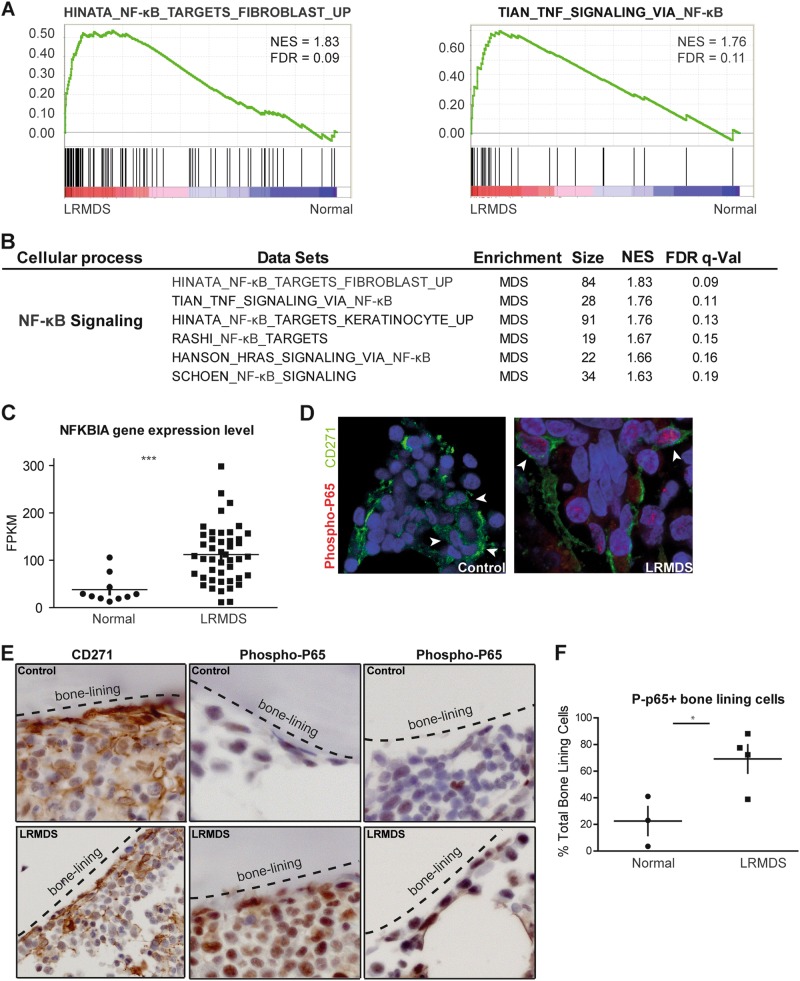
Activation of NF-κB-mediated signaling in LR-MDS mesenchymal cells. **a** Representative GSEA plots demonstrating activation of NF-κB signaling in mesenchymal cells from LR- MDS. **b** Summary of gene sets implicating NF-κB activation in mesenchymal cells from 45 LR-MDS patients. Gene set size, NES, and FDR value of each gene set are as listed. GSEA gene sets enrichment analysis, NES normalized enrichment score, FDR false discovery rate. **c** Gene expression level (in FPKM) of *NFKBIA* in normal and LR-MDS samples. **d** Representative images showing immunofluorescence staining of CD271 and phospho-p65 in both age-matched control (left panel) and LR-MDS patient (right panel) bone marrow slides confirming activation of NF-κB in mesenchymal cells. The white arrow indicates the absence or presence of nuclear phospho-p65 signal (red) in CD271^+^ (green) mesenchymal cells. The nuclei were counterstained with DAPI. **e** Representative photomicrographs of the distribution of CD271^+^ mesenchymal cells (left panels). These cells are enriched at the endosteal surface (marked by bone-lining area) and have a spindle-shaped morphology. Representative immuno-histochemical analysis of phospho-p65 (middle and right panels) in age-matched controls (top) and LR-MDS (bottom) patients, demonstrating NF-κB activation in spindle-shaped endosteal cells in LR-MDS. **f** The percentage of phospho-p65+ bone-lining cells as a fraction of the total bone-lining cells in LR-MDS (*n* = 4) compared to age-matched controls (*n* = 3). ***P < .001, *P < .05. FPKM fragments per kilobase of exon per million fragments mapped; *NFKBIA* NF-kappa-B inhibitor alpha

While the genes encoding inflammatory factors and negative regulators of hematopoiesis [4] are bona fide downstream targets of NF-κB signaling in other experimental settings, we next wanted to provide experimental support for the view that NF-κB activation specifically in mesenchymal precursor cells results in upregulation of these targets. To this end, we designed a strategy of activating NF-κB signaling in mesenchymal progenitor cells by stably overexpressing the constitutively active form of IKK2 (FLAG-IKK2SE), a kinase upstream regulator of NF-κB, via a lentiviral vector (Fig. 2a, Figure S3A-C) [7] in OP9 cells. OP9 cells, like CD271^+^ cells [4], express osteolineage commitment markers as well as HSPC regulatory factors and robustly support the expansion of human HSPCs [8]. NF-κB activation in OP9 cells resulted in overexpression of *NFKBIA* (Fig. 2c) and canonical NF-κB downstream negative regulators of hematopoiesis, including *Il6*, *Cxcl2* (murine homolog of *IL8*), *Ccl3*, *Inhba*, *Fth1*, *Ltf*, *Ccl5*, *and Cxcl4* (Fig. 2b), recapitulating the findings in LR-MDS patients. Similar to the results in OP9 cells, activation of NF-κB in human mesenchymal cells (HS5 cell line and expanded bone-marrow-derived primary mesenchymal cells) (Figure S3D) also resulted in upregulation of NF-κB downstream targets including negative regulators of hematopoiesis such as *IL6, IL8, CCL3, S100A9, INHBA*, and *CCL5* (Figure S3E). Together, the data link the transcriptional landscape of inflammatory alterations in mesenchymal cells to activation of NF-κB in LR-MDS.

**Fig. 2:**
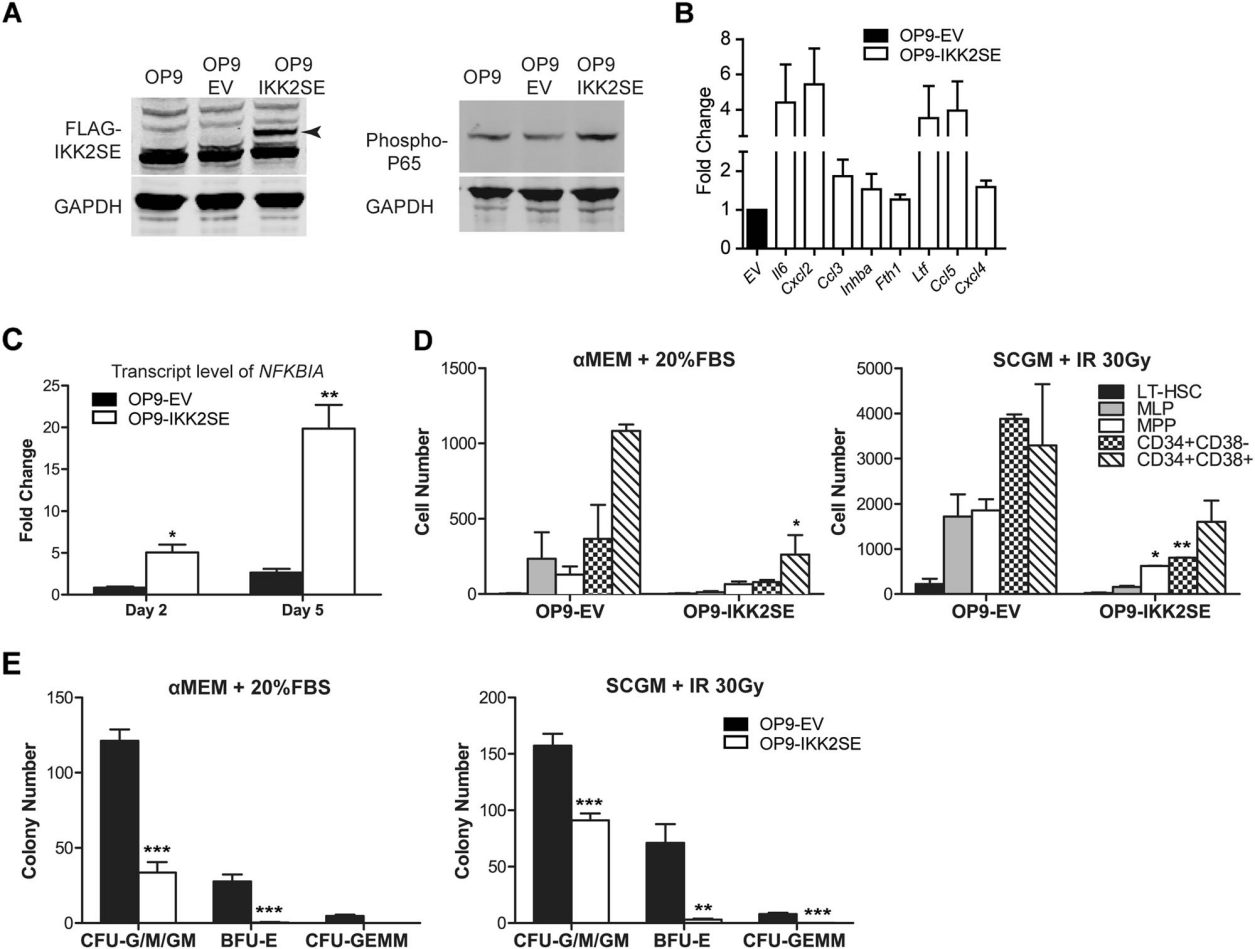
Activated NF-κB signaling in mesenchymal cells attenuates HSPC number and function. **a** Western blot analysis showing the overexpression of Flag-IKK2SE and nuclear phospho-p65, the phosphorylated (active) form of NF-κB, in IKK2SE transduced OP9 cells in comparison to empty vector (EV)-transduced or wild-type cells. **b** Expression level of NF-κB downstream targets (*Il6, Cxcl*2) and disease-relevant negative regulators of hematopoiesis [3, 4] in OP9 cells transduced with IKK2SE. Fold change relative to EV is presented (*n* = 3 for each transcript). **c** Expression level of *NFKBIA* in OP9 cells transduced with EV or IKK2SE and re-plated in serum-containing medium for 2 or 5 days. Fold change relative to wildtype OP9 cells is presented (*n* = 3). **d** Co-culture experiments with bone marrow CD34^+^ HSPCs and OP9 stromal cells transduced with either EV or IKK2SE. The co-culture took place for 7 days before analysis. The absolute number of immunophenotypically defined HSPC subsets (Lin^−^CD34^+^CD38^+^ progenitors, Lin^−^CD34^+^CD38^−^ HSPCs, Lin^−^CD34^+^CD38^−^CD45RA^−^CD90^+^ LT-HSCs, Lin^−^CD34^+^CD38^−^CD45RA^−^CD90^−^ MPPs, Lin^−^CD34^+^CD38^−^CD45RA^+^CD90^−^ MLPs) on day 7 after co-culturing with OP9-EV or OP9-IKK2SE, in serum-containing medium condition (left panel) and serum-free, stem cell growth medium (SCGM) condition with OP9-EV and OP9-IKK2SE irradiated at 30 Gy (right panel). Data represent mean±SEM of two independent experiments. **e** The total number of CFU-C after 7 days co-culture in serum-containing medium condition (left panel) and serum-free medium condition with OP9-EV and OP9-IKK2SE irradiated at 30 Gy (right panel). Data represent mean±SEM of two independent experiments performed in triplicate. Unpaired *t*-test was performed for statistical analysis; ***P < .001, **P < .01, *P < .05

As earlier reported, in human MDS, the majority of CD34^+^ HSPCs is in direct contact with CD271^+^ mesenchymal cells [9]. To assess the effect of NF-κB activation in mesenchymal cells on the biology of normal HSPCs, we performed co-culture experiments with bone marrow CD34^+^ HSPCs and OP9 cells transduced with IKK2SE or empty vector (EV) (Fig. 2d). Co-culture for 7 days on IKK2SE-transduced OP9 cells resulted in significantly reduced numbers of immunophenotypically defined HSPCs in comparison to EV-transduced mesenchymal cells (Fig. 2d). In addition, reduced HSPC number was reflected in a reduced number of CFU-Cs (Fig. 2e), indicating attenuation of progenitor function in this setting. NF-κB activation resulted in impaired proliferation of mesenchymal cells in serum-containing culture conditions (Figure S4A) which may contribute to the reduced support for HSPCs in vitro (Fig. 2d, left panel). However, equalizing mesenchymal cell numbers by arresting their proliferation (using irradiation and maintenance in non-proliferative, serum free, culture conditions) (Figure S4B) recapitulated the significantly reduced numbers of immunophenotypically defined HSPCs on IKK2SE-transduced OP9 cells in comparison to EV-transduced mesenchymal cells (Fig. 2d, right panel). This included the fraction enriched for long-term hematopoietic stem cells (LT-HSCs) defined by the markers CD34^+^CD38^−^CD45RA^−^CD90^+^, multipotent progenitor (MPP; CD34^+^CD38^−^CD45RA^−^CD90^−^), multilymphoid progenitor (MLP; CD34^+^CD38^−^CD45RA^+^CD90^−^), as well as primitive (CD34^+^CD38^−^) and committed CD34^+^CD38^+^ progenitor cells (Fig. 2d), indicating that mesenchymal NF-κB signaling attenuates HSPC number and function, at least partially, independent of its effect on mesenchymal cell proliferation.

Collectively, in this brief communication, we demonstrate that mesenchymal NF-κB activation is a common finding in LR-MDS patients leading to transcriptional upregulation of inflammatory programs associated with negative regulation of hematopoiesis and attenuation of HSPC numbers and function.

Demonstration of mesenchymal activation of NF-κB provides human disease relevance to a number of murine studies implicating NF-κB activation in ancillary cells to the pathogenesis of hematopoietic disease. NF-κB activation in non-hematopoietic cells has been shown to induce ‘MDS-like’ myeloproliferative disease (MPD) in mice [10]. In another study, NF-κB activation in bone marrow mesenchymal cells and endothelial cells, as a result of elevated levels of the microRNA miR-155, generated a persistent pro-inflammatory state of the bone marrow niche leading to an MPD-like disease in a Notch/RBPJ loss-of-function mouse model [11]. Our experimental data demonstrate that NF-κB activation in the mesenchyme attenuates normal hematopoiesis, which is of key relevance to LR-MDS characterized by cytopenia. The implication of mesenchymal NF-κB activation in the pathogenesis of MDS may also point toward common mechanisms between the pathogenesis of MDS and oncogenesis in other systems. NF-κB-mediated chronic tissue inflammation has been shown to drive cancer initiation and progression via secretion of cytokines and soluble factors in models of several other forms of cancer [12, 13], including skin, prostate, and colon cancer. In these models, activation of NF-κB signaling, specifically in fibroblasts, promoted malignant features in heterotypic (pre)cancerous cells, supporting the hypothesis that mesenchymal inflammation may facilitate tumorigenesis in the hematopoietic system as well.

The activation of NF-κB signaling in mesenchymal cells in most LR-MDS patients raises intriguing questions about the events driving this activation. This includes the question whether mesenchymal cell-intrinsic alterations or extrinsic events are driving NF-κB activation. While the answer to this question remains speculative in the absence of experimental evidence (and may vary between patients), it is conceivable that primary alterations in hematopoietic elements drive activation of NF-κB activation in the mesenchymal niche.

This notion is supported by recent findings where activation of the NF-κB pathway in CD34^+^ HSPCs is implicated in MDS pathogenesis [14] and CD271^+^ mesenchymal cells co-localize with CD34^+^ HSPCs in the bone marrow section of MDS patients [9]. It is therefore reasonable to hypothesize that activated NF-κB pathway in HSPCs signals to the adjacent mesenchymal elements, resulting in NF-κB activation in mesenchymal cells. As NF-κB activation is likely maintained through autocrine/paracrine feedback signaling networks, other cellular types that anatomically localize with the activated HSPCs and mesenchymal elements could be involved as well, suggesting that diverse cellular components may participate in this crosstalk. The combined findings suggest that in LR-MDS, activation of NF-κB occurs in both hematopoietic and mesenchymal cells, likely through autocrine and paracrine feedback signaling networks, leading to a NF-κB-mediated inflammatory milieu in the LR-MDS bone marrow and an overexpression of a repertoire of secreted negative hematopoietic regulators. S100A8/9, recently shown by us to induce NF-κB activation and genotoxic stress in HSPCs and to be associated with an increased likelihood of leukemic transformation, was not correlated with *NFKBIA* expression in patients, indicating that its regulation is more complex and may include upstream TP53 activation [3].

Taken together, the findings support the notion that mesenchymal factors, in addition to hematopoietic cell autonomous characteristics, may be therapeutically targeted in LR-MDS and warrant ongoing experiments defining the contribution of NF-κB activation and inflammation to ineffective hematopoiesis and leukemic evolution in MDS.

## Electronic supplementary material

Supplementary information
