# Supplementary material for: Activation of NF-κB driven inflammatory programs in mesenchymal elements attenuates hematopoiesis in low-risk myelodysplastic syndromes
Source: Leukemia. 2018 Oct 12;33(2):536–41. doi: 10.1038/s41375-018-0267-x (PMC6365382; doi:10.1038/s41375-018-0267-x)
Supplement: Supplementary file 1 — Supplementary information [file 41375_2018_267_MOESM1_ESM.docx]

**Supplementary Information**

**Supplementary material and methods**

**Patient and healthy donor bone marrow samples**

Bone marrow samples from LR-MDS patients and healthy donors were obtained as previously described, including the clinical characteristics of the patients and information about the healthy donors.^1, 2^ In short, all 45 patients were included in the HOVON89 clinical trial (HOVON89; www.hovon.nl; www.trialregister.nl as NTR1825; EudraCT No. 2008-002195-10) where patients with low or intermediate-1 risk MDS based on IPSS criteria were recruited. The healthy controls were obtained from donors for allogeneic transplantation. All samples were collected with informed consent, approved by the Institutional Review Board of the Erasmus Medical Center, the Netherlands, in accordance with the declaration of Helsinki.

**Flow cytometry analysis**

Sorting of the mesenchymal cells from the bone marrow of patients and normal donors were performed by FACS as previously described.^1, 2^ The cells were directly sorted in 800μl Trizol (Ambion) for RNA isolation.

For immunophenotyping of CD34+ HSPCs after 7 days of co-culture, the following antibodies were used based on earlier work with optimized dilutions: Lin-APC (1:25), CD34-PE-Cy7 (1:30), CD38-PercP-Cy5.5 (1:60), CD90-PE (1:30) and CD45RA-APC-H7 (1:30).^3^ Live cells were selected based on the DAPI (1:7500) gate. The gating strategy defining the different subsets of HSPCs is illustrated in supplemental figure 3F-G. The OP9 stromal cells were excluded based on ZsGreen expression, as the transduced OP9 cells were ZsGreen (GFP) positive (Figure S3C). The data was acquired using a LSRII flow cytometer (BD Biosciences) and analyzed using FlowJo software.

Counting of the cells after 7 days of co-culture was accurately obtained with flow-count fluorosphere beads (Beckman Coulter), in combination with the following antibodies: CD34-PE-Cy7 (1:50) and CD45-BV510 (1:50). The gating strategy is shown in Figure S3H. The data was acquired using a LSRII flow cytometer (BD Biosciences) and analyzed using FlowJo software. The number of cells was calculated based on the following formula: CD34+ cells per μl = (Live CD34+ events x bead concentration)/number of acquired single beads/(volume beads/volume cells). The number of total mononuclear cells (MNCs) determined by cell counting with beads and frequency of each HSPCs subpopulation from immunophenotyping were combined to obtain the number of cells present in individual subpopulations.

**RNA extraction and RNA quality control**

Total sample RNA isolation was performed according to the standard protocol of RNA isolation with Trizol and GenElute LPA (Sigma). In the end, the RNA pellet was resuspended in 7.5μl of RNAse free water (Qiagen) and quality and quantity of the total RNA was checked on a 2100 Bio-analyzer (Agilent) using the Agilent RNA 6000 Pico Kit.

**RNA sequencing and gene expression profiling**

RNA sequencing and the downstream data processing were performed as previously described.^1, 2^ In short, SMARTer Ultra Low RNA Kit (Clonetech) for Illumina Sequencing was used to prepare the cDNA based on the manufacture’s protocol. Once the cDNAs were obtained, the subsequent library preparation steps, sequencing and alignments were performed as earlier described, using a range of tools including the cutadapt program, TopHat2 and cufflinks.^4^ The resulting gene expression values were measured as FPKM (Fragments per kilobase of exon per million fragments mapped). Fragment counts were determined per gene with HTSeq-count, utilizing the strict intersection option, and subsequently used for differential expression analysis using the DESeq2 package, with standard parameters, in the R environment. Multiple testing correction was performed with the Benjamini-Hochberg procedure to control the False Discovery Rate (FDR). Finally, gene set enrichment analysis (GSEA) was performed on the FPKM values using the curated C2 collection of gene sets within MSigDB.^5^

Direct comparison between gene expression data from purified and *ex vivo* expanded LR-MDS mesenchymal cells was performed as previously described.^2^ In brief, BAM files containing the molecular data of *ex vivo* expanded stromal cells derived from LR-MDS patients (n=5)^6^ were obtained from the European Genome-Phenome Archive data base (EGAS00001000716). GSEA was then performed comparing our gene expression data of the purified LR-MDS mesenchymal cells (n=45) to the gene expression data of expanded stromal cells (n=5).^5^

**Immunohistochemistry and immunofluorescence**

Immunohistochemical and immunofluorescence stainings of LR-MDS and control paraffin-embedded bone marrow slides were performed as previously described.^7^ CD271 and phospho-p65 antibodies were used to mark the stromal cells of interest with their corresponding phospho-p65 status. Immunohistochemical single staining facilitated the enumeration of phospho-p65^+^ bone lining stromal cells; whereas the immunofluorescence double staining demonstrated the co-localization of CD271^+^ and phospho-p65^+^ cells in patient and control sections.

All the bone marrow sections (5-µm) were deparaffinized in xylene and hydrated in a graded series of alcohol. Antigen retrieval was achieved by microwave treatment in citrate buffer (10mM pH 6.0) and blocking of the endogenous peroxidases was performed with 3% H_2_O_2_ in PBS. For IHC staining, sections were blocked using 10% normal human and goat serum (DAKO) in Teng-T solution (10 mM Tris, 5 mM EDTA, 0.15 M SodiumCl, 0.25 % gelatine, 0.05 % Tween-20) followed by an overnight incubation at 4°C with primary antibody: rabbit anti-human phospho Serine 276 of p65 (p-p65, active form; Santa Cruz Biotechnology) diluted 1:400, CD271 (Sigma Aldrich) diluted 1:200, or normal rabbit immunoglobulin (DAKO) diluted accordingly. Immunoreactions were detected using biotinylated secondary antibody (goat anti-rabbit, 1:2000 dilution) with Vectastain ABC Elite Kit (Vector Laboratories) and 3,3’-diaminobenzidine tetrahydrochloride (Sigma Aldrich). For all stainings, nuclei were counterstained with haematoxylin (Vector Laboratories). Images of stained tissues were acquired using a Leica DM5500B upright microscope with 40x lenses and LAS-AF image acquisition software (Leica). The number of phospho-p65+ bone lining cells were manually quantified using ImageJ software from x40 photomicrographs of blindly selected trabecular bone area. Unpaired t-test was performed and *p* < .05 was considered significant.

For IF staining, sections were blocked with 10% normal human, goat and horse serum (DAKO) in Teng-T solution followed by an overnight incubation at 4°C with a primary antibody directed against phospho Serine 276 of p65. The next day, biotinylated goat anti-rabbit secondary antibody and streptavidin-Dylight 594 (1:200, Vector Laboratories) were incubated consecutively. This was followed by an additional incubation with a primary mouse anti-human antibody directed against CD271 (1:100, eBioscience) followed by conjugated horse anti-mouse Dylight 488 (1:200, Vector Laboratories). Counterstain with DAPI and mounting were performed using Vectashield mounting medium (Vector Laboratories, H-1200). Control slides with single stains for the primary antibodies CD271 or phospho-p65 combined with all conjugated secondary antibodies were performed as a background control. Images were acquired on a Leica SP5 confocal laser scan microscope equipped with Diode/Argon/HeNe lasers using a 40x planapochromat oil immersion objective. Images were analyzed using ImageJ software.

**Lentiviral transduction of OP9, HS5 and primary expanded stromal cells**

The plasmid containing mutant form of IKK-2 (FlagIKK-2 S177E S181E; referred hereafter as IKK2SE), which has previously been demonstrated to result in constitutive NF-κB activation, was obtained from Addgene.^8, 9^ The Flag-IKK2SE fragment was recloned into the pHAGE ires ZsGreen lentiviral vector (kindly provided by Dr. R. Delwel). Stable transduction of OP9 cells (ATCC, CRL-2749) with pHAGE-EV and IKK2SE was performed and transduction efficiency was determined by FACS measuring ZsGreen expression of the cells (Figure S3C). Overexpression of IKK2SE was confirmed by Western blotting against Flag-tag and the subsequent activation of NF-κB was verified using an antibody against the active form of NF-κB: phospho-p65 (Ser 536; Cell Signaling).

Lentivirus for both pHage-EV or pHage IKK2SE (Flag-tagged IKK2 S177E S181E) was produced in 293T cells, by means of transient transfection of the pHage EV or pHage IKK2SE, together with the packaging plasmids pSPAX and pMD2.G. OP9 cells were transduced with equal titer lentivirus and 3 days later OP9-EV or OP9-IKK2SE were used for subsequent co-culture experiments.

The same transduction procedures were carried out for human HS5 stromal cell line (ATCC, CRL-11882) and primary expanded stromal cells. All cell lines used in this study were authenticated and mycoplasma free; all cell lines were maintained in cell culture medium, consist of Dulbecco's Modified Eagle Medium (DMEM, Life Technologies) supplied with 10% fetal bovine serum (FBS) and 1% penicillin/streptomycin.

**Luciferase Reporter Assay**

We performed NF-κB responsive luciferase reporter assays to test the functionality of the recloned pHage IKK2SE construct. HEK293T cells were transiently co-transfected with a luciferase vector containing three NF-κB responsive elements together with either pHage EV or pHage IKK2SE. Cells were harvested after 48 hours and luciferase activity was measured with the Dual-Luciferase Reporter Assay System (Promega) using a Victor3 plate reader (PerkinElmer). As a control for the luciferase assay pMIG EV and pMIG HA-IKK2SE were used (Figure S3A and S3B). Luciferase vector containing 3x NF-κB responsive elements and pMIG HA-IKK2SE were kindly provided by Dr. H. Schepers and J.J. Schuringa.^10^

**Western Blotting**

Protein extracts were made by lysing cells in Carin lysis buffer (20 mM Tris-HCl pH 8.0, 138 mM NaCl, 10mM EDTA, 100 mM NaF, 1% Nonidet P-40, 10% glycerol, 2mM NA-vanadate) supplemented with 0.5 mM DTT and the protease inhibitor SigmaFast (Sigma Aldrich). Equal amounts of proteins were denatured and separated on a Novex NuPage 4-12% Bis-Tris Gradient gel (Life Technologies) and transferred to Protran BA83 blotting paper (GE Healthcare Life Sciences). Primary antibodies mouse anti-Flag (1:1000) (Sigma Aldrich) or Rabbit anti-phospho p65 S536 (1:1000) (Cell Signaling) were incubated overnight at 4°C after necessary blocking step. Flag antibody was used to confirm transfection or transduction efficiency; whereas phospho-P65 was used to demonstrate phospho-p65 overexpression in IKK2SE transduced cells. As secondary antibodies (1:10000 for both) donkey anti-mouse 800 or goat anti-rabbit 800 (Li-COR Biosciences) were used. GAPDH (Santa Cruz Biotechnologies) was used as a loading control (primary: rat anti-GAPDH (1:1000), secondary: donkey anti-rat 680 (1:10000). After the final incubation, Western blots were scanned and processed using an Odyssey Infrared Imager (Li-COR Biosciences).

**Quantitative PCR**

Total RNA of OP9-EV, OP9-IKK2SE, HS5-EV, HS5-IKK2SE, primary stroma-EV, primary stroma-IKK2SE cells was isolated 3 days after transduction, or at time points as indicated otherwise, using Trizol reagent (Ambion), followed by cDNA synthesis using SuperScript II Reverse Transcriptase II kit (Invitrogen). Q-PCR was performed using Fast SYBR Green master mix (Applied Biosystems) on a 7500 Fast Real-Time PCR system (Applied Biosystems) with the following primer sets:

Murine: *Il6*: Fw 5'-TCGGAGGCTTAATTACACA-3' Rv 5'-CTGGCTTTGTCTTTCTTGTT-3; *Cxcl2* (murine homologue of IL8): Fw 5'-GCGCCCAGACAGAAGT-3' Rv 5'-CGGGTGCTGTTTGTTTT-3'; *Ccl3*: Fw 5’-TTCTCTGTACCATGACACTCTGC-3’ Rv 5’-CGTGGAATCTTCCGGCTGTAG-3’; *Inhba*: Fw 5’-TCACCATCCGTCTATTTCAGCA-3’ Rv 5’-CTTCCGAGCATCAACTACTTTCT-3’; *Nfkbia*: Fw 5’-TGAAGGACGAGGAGTACGAGC-3’ Rv 5’-TTCGTGGATGATTGCCAAGTG-3’; *Fth1*: Fw 5’-CAAGTGCGCCAGAACTACCA-3’ Rv 5’-GCCACATCATCTCGGTCAAAA-3’; *Ltf*: Fw 5’-TGAGGCCCTTGGACTCTGT-3’ Rv 5’-ACCCACTTTTCTCATCTCGTTC-3’ *Ccl5*: Fw 5’-GCTGCTTTGCCTACCTCTCC-3’ Rv 5’-TCGAGTGACAAACACGACTGC-3’; validated all-in-one murine C*xcl4* (PF4) primers (Genecopoeia).

Human*: IL6*: Fw 5’-CCCCCAGGAGAAGATTC-3’ Rv 5’- GCTGCTTTCACACATGTTACT-3’; *IL8*: Fw 5’- CCGGAAGGAACCATCT-3’ Rv 5’-TTGGGGTGGAAAGGTT-3’; *CCL3*: Fw 5′-GCAACCAGTTCTCTGCATCA-3′ Rv 5′-TGGCTGCTCGTCTCAAAGTA-3′; *INHBA*: Fw 5’-ACGGGTATGTGGAGATAGAGGA-3’ Rv 5’- GGACTTTTAGGAAGAGCCAGACT-3’; *CCL5*: Fw 5’-CGCTGTCATCCTCATTGCTA-3’ Rv 5’-CCATTTCTTCTCTGGGTTGG-3’; *S100A9*: Fw 5’-TTCAAAGAGCTGGTGCGAAAAG-3’ Rv 5’-GCATTTGTGTCCAGGTCCTCC-3’; *Ikk2SE*: Fw 5’-GGTGAGCAGATTGCCATCAAG-3’ Rv 5’- ACCCTCAGTTCGCTGGTCTCG-3’

The expression level of each molecule was normalized against the housekeeping gene *Hprt* (in OP9) or *GAPDH* (in HS5 or primary expanded stromal cells) and the fold change of the expression level of each gene in IKK2SE-transduced cells was calculated relative to EV transduced cells or to wildtype OP9 cells as indicated.

**Isolation and culture of bone marrow CD34+ cells**

Human bone marrow CD34^+^ cells (obtained from healthy donors) were selected by MiniMacs (Miltenyi Biotec) followed by LS and MS MACS separation columns (Miltenyi Biotec) to achieve optimal purity. In the co-culture setting, 38000 OP9-EV or OP9-IKK2SE cells per 1.9cm^2^ were plated in cell culture medium one night before the experiment. The next morning, where applicable, irradiation of OP9-EV or OP9-IKK2SE monolayers was performed in RS320 RS X‐Ray Cabinet (Xstrahl) at a single dosage of 30 Gray (Gy). Subsequently 15000 CD34^+^ cells per 1.9cm^2^ were resuspended in either serum containing medium or serum free medium, and directly plated on either a OP9-EV or a OP9-IKK2SE monolayer (Figure 2D). Serum containing culture medium consisted of ɑMEM (GE Healthcare Life Sciences‎) supplemented with 20% FBS, Serum free culture medium consisted of GMP serum-free Stem Cell Growth Medium (SCGM, CellGenix); both media were further supplied with 1% penicillin/streptomycin, 50 ng/ml stem cell factor (SCF), 50 ng/ml FLT3 and 50 ng/ml thrombopoietin. Cultures were kept at 37°C in a 5% CO_2_ incubator for 7 days until further analysis.

**CFU-C**

On day 7 of the co-culture, 4000 mononuclear cells (MNCs) from OP9-EV condition, and equal volume of cells in OP9-IKK2SE condition (applicable to both serum containing and serum free conditions) were collected and resuspended in IMDM medium. This cell suspension was added to MethoCult™ GF H84434 (StemCell Technologies), which allows the growth of colonies from all three lineages, and triplicate dishes were plated. The methocult plates were kept at 37°C in a 5% CO_2_ incubator for 2 weeks until colony counting.

**Correlation plots**

Correlation plots for the specified genes were based on FPKM values, obtained from our previous work. ^1, 2^ Non-parametric correlation estimates (Spearman’s rho) and corresponding two-sided p-values were calculated using the cor.test() function available in base R (version 3.4.1) on Windows 10. The FDR was used to adjust P-values for multiple testing. FDR-corrected p-values smaller or equal to 0.05 were considered statistically significant.

**Clinical outcome of LR-MDS patients**

Overall survival curves were calculated using the survival R package. Alive patients were censored at final follow-up and deaths from any cause were considered events. The proportional hazard assumption was visually inspected using (-log(-log(S(t)))) plots. The Peto & Peto modified Gehan-Wilcoxon test was used to test for statistically significant differences in the distribution of survival times between *NFKBIA* subgroups (P-values ≤ 0.05 were considered statistically significant). To investigate the possible differences in progression-free survival in both *NFKBIA* subgroups, we calculated the cause-specific cumulative incidence probabilities for progression of disease (defined as an increase in blasts, either in bone marrow or in peripheral blood [see Appendix C of the HOVON89 clinical trial protocol], or development of leukaemia), while accounting for competing risks. To this end, we made use of the cuminc() function from the cmprsk R package. We tested for differences in cumulative incidence probabilities with Gray’s test, implemented in the cuminc() function of the cmprsk R package. Visual representations of the data were obtained with the help of the ggplot2, ggfortify and survminer R packages for survival data.

**Supplementary Figures**


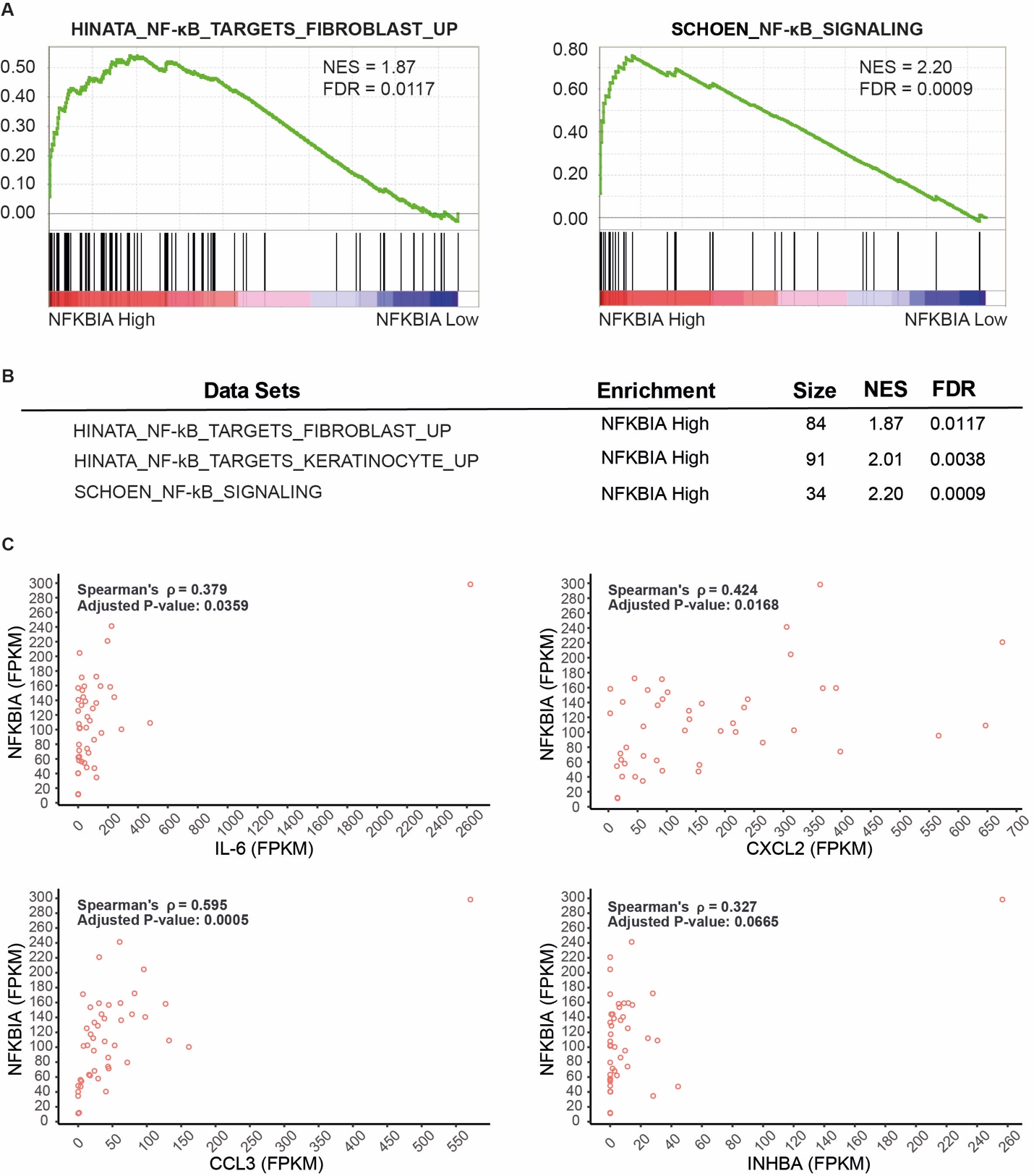


**Supplemental Figure 1. Activation of NF-κB signaling in mesenchymal niche cells in a subset of LR-MDS patients.** (A) Representative GSEA plots demonstrating enrichment of NF-κB signatures in patients with high (*NFKBIA*^+^) expression (see also figure 1C) in purified mesenchymal cells. The *NFKBIA* cut-off level was arbitrarily defined as mean + 1SD of normal (FPKM 67,93). Other cut-off levels (FPKM 50 or FPKM 107) yielded similar results (data not shown). (B) Listing of enriched NF-κB signatures in *NFKBIA*+ patients. NES: normalized enrichment score; FDR: False Discovery Rate. (C) Expression of *NFKBIA* is significantly correlated to expression of downstream canonical transcriptional targets of NF-κB signaling. Non-parametric correlation estimates (Spearman’s rho) and two-sided p-values (α = 0,05) for the relationship between *NFKBIA* expression and *IL6*, *CXCL2*, *CCL3* and *INHBA* are shown. Correlations were obtained using raw FPKM values. P-values were adjusted for multiple testing using the FDR.


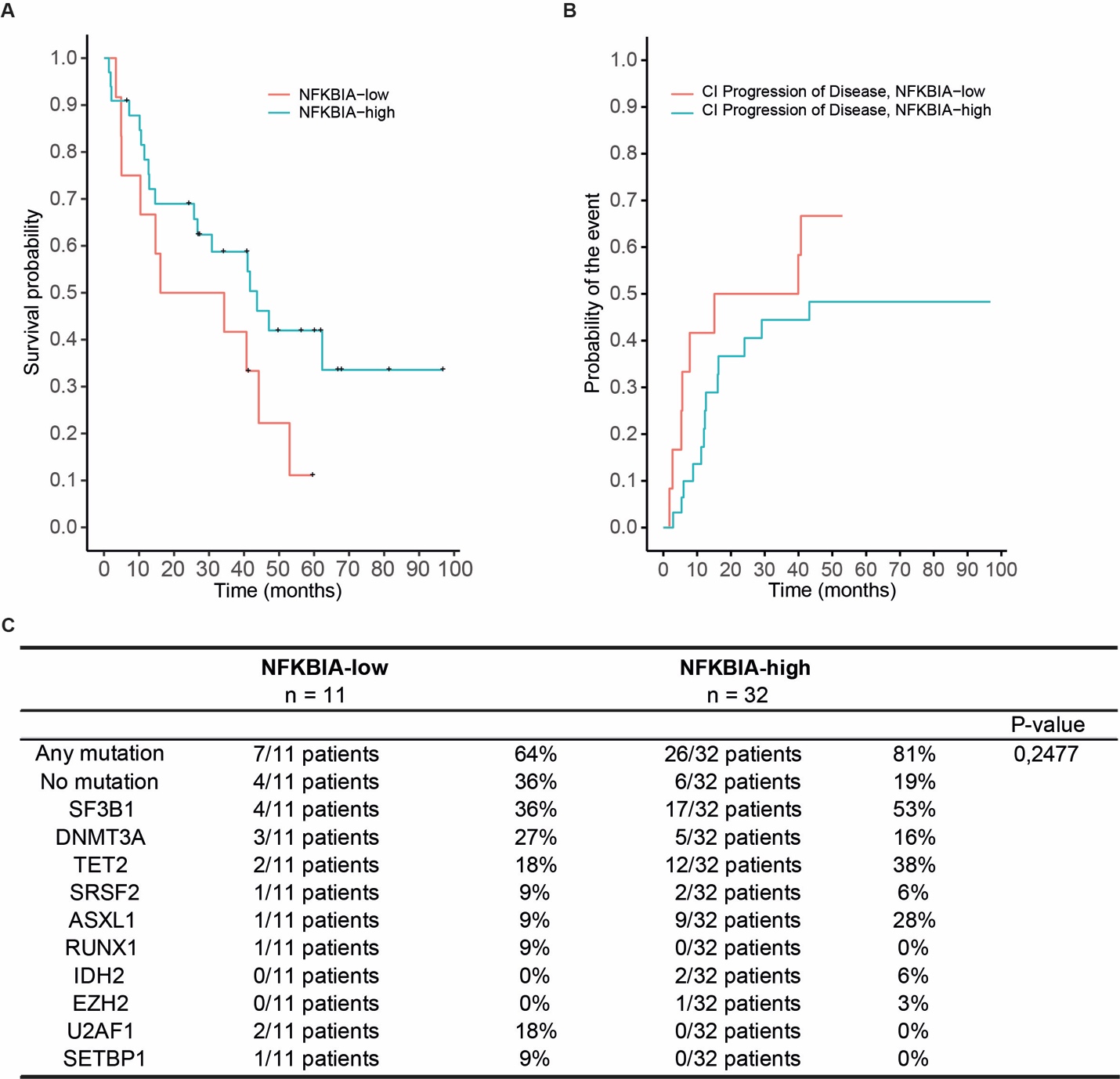


**Supplemental Figure 2. Correlation of activated NF-κB signaling in niche cells to clinical outcome and mutational status in LR-MDS.** (A) Overall survival probabilities stratified on *NFKBIA* gene expression (low (n = 12) vs. high (n = 33)) were obtained by calculating the Kaplan-Meier estimator. (B) Cumulative incidence curves for progression of disease, stratified on *NFKBIA* low (n = 12) vs. high (n = 31). For two patients, no data was available for the cumulative incidence curves (total n = 43). (C) Mutational status as assessed by gene-panel sequencing. Fisher's Exact Test was used to investigate the difference in proportion of mutations (any vs. no mutation) between *NFKBIA*-low and *NFKBIA*-high (OR = 0.414; 95% CI [0,0713; 2,562]; P = 0.2477). One patient key was unavailable for the *NFKBIA*-low group (total n = 12); one patient of the *NFKBIA*-high group was not sequenced (total n = 33).


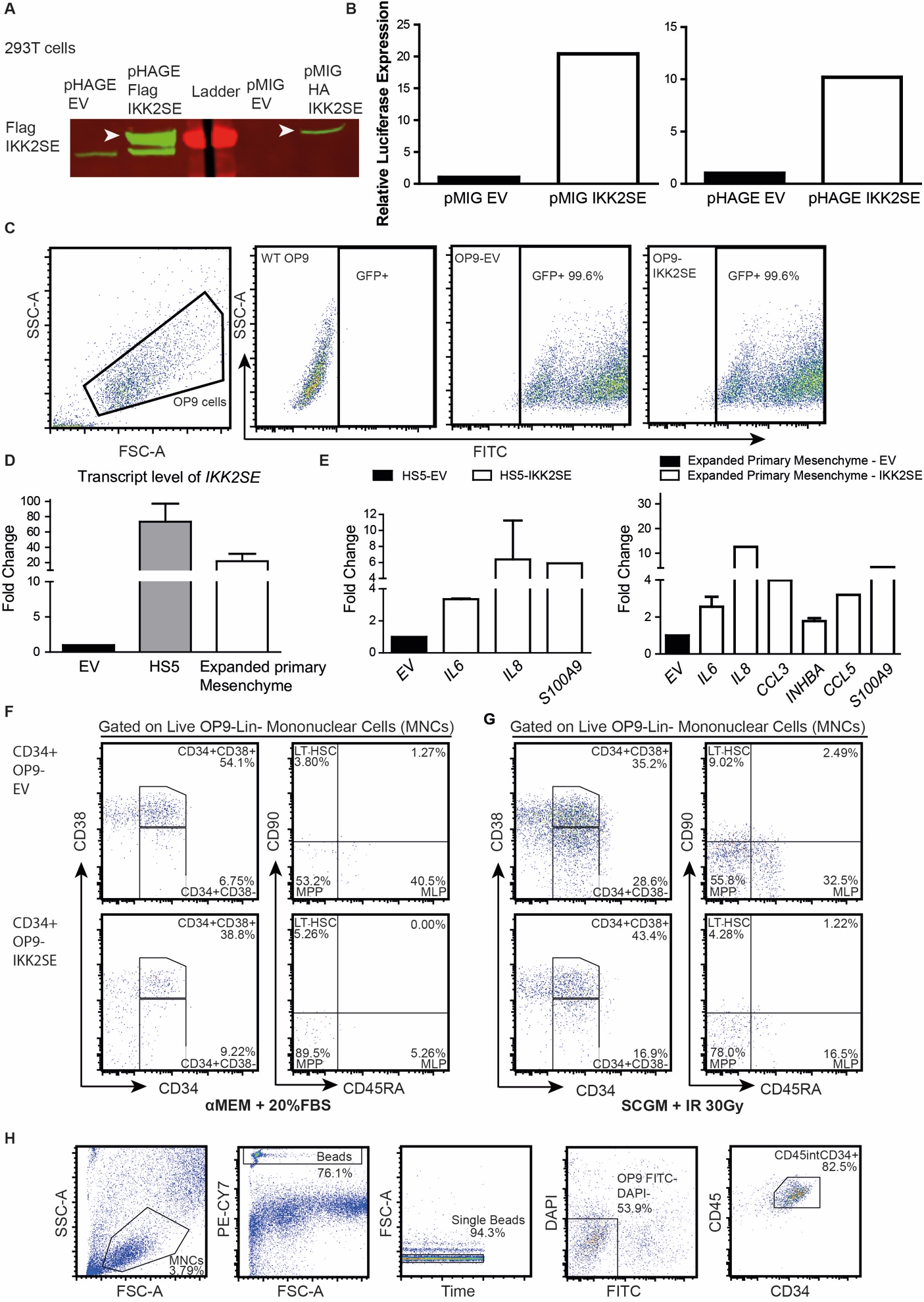


**Supplemental Figure 3. Activation of NF-κB signaling in OP9, HS5 and expanded primary stromal cells.** (A) Western blot analysis showing the overexpression of Flag-IKK2SE transfected 293T cells in comparison to pHage-empty vector (EV) transfected 293T cells. As a reference for the protein size of Flag-IKK2SE, lysates of pMIG EV and pMIG HA-IKK2SE transfected 293T cells were included. (B) Level of NF-κB luciferase response in pHage-EV and pHage-Flag IKK2 transfected 293T cells (right panel). NF-κB luciferase response in pMIG EV and pMIG HA-IKK2SE transfected 293T cells were included as a positive control. (C) Transduction efficiency of phage EV and pHage-Flag IKK2SE in OP9 cells measured by ZsGreen expression comparing to non-transfected OP9 cells. (D) Expression level of IKK2SE in HS5 and expanded primary mesenchyme transduced with either EV or IKK2SE. (E) Expression level of NF-kB downstream targets and disease-relevant negative regulators of hematopoiesis in HS5 mesenchymal cell lines (left panel) and expanded primary mesenchyme (right panel) transduced with EV or IKK2SE. Fold change relative to EV is presented. (F-G) Representative FACS plots showing the immunophenotypically defined HSPCs subsets after exposure to OP9-EV/IKK2SE in serum containing medium condition (F) and serum free medium condition with stromal layers irradiated at 30 Gy (G). The presented plots were gated on live/OP9-/Lin- mononuclear cells (MNCs). The HSPCs subfractions are defined as: CD34^+^CD38^+^ progenitor population, CD34^+^CD38^-^ HSPCs, CD34^+^CD38^-^CD45RA^+^CD90^-^ MLPs (multilymphoid progenitors), CD34^+^CD38^-^CD45RA^-^CD90^-^ MPPs (multipotent progenitors), and CD34^+^CD38^-^CD45RA^-^CD90^+^ LT-HSCs (long-term hematopoietic stem cells). (H) Representative FACS plot demonstrating the cell counting approach by flow-count fluorosphere beads. Information concerning total number of beads and MNCs were obtained during acquiring on LSRII. OP9 cells were gated out from the MNCs by ZsGreen expression and live cells were selected by DAPI staining. CD45 and CD34 antibodies were applied to identify the frequency of CD34^+^ HSPCs.


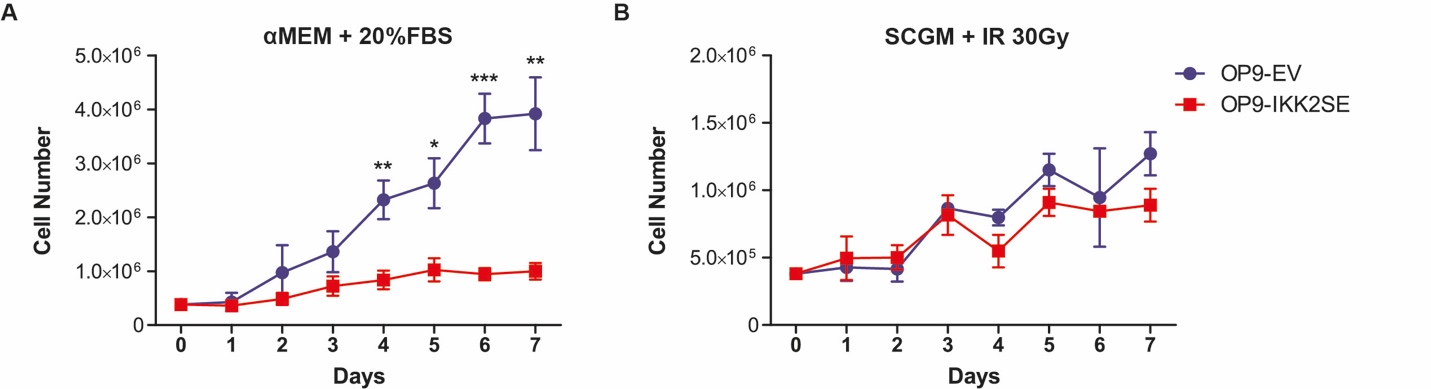


**Supplemental Figure 4. Mesenchymal cell numbers upon activation of NF-κB signaling in different culture conditions.** (A) Proliferation curve of OP9-EV/IKK2SE cells in serum containing medium condition (n=4). (B) Proliferation curve of OP9-EV/IKK2SE cells in serum free medium with 30 Gy irradiation condition (n=2). OP9 cells transduced with EV or IKK2SE were re-plated in 24-well plates under conditions described above for 7 days. Where applicable, irradiation was performed at day 0 following re-plating. Each day OP9-EV/IKK2SE cells in corresponding wells were trypsinized, subsequently total cell number was quantified and plotted. Unpaired t-test was performed for statistical analysis *** *P* < .001 ** *P* < .01 * *P* < .05

**Supplementary References**

1. Zambetti NA, Ping Z, Chen S, Kenswil KJ, Mylona MA, Sanders MA*, et al.* Mesenchymal Inflammation Drives Genotoxic Stress in Hematopoietic Stem Cells and Predicts Disease Evolution in Human Pre-leukemia. *Cell Stem Cell* 2016 Nov 3; **19**(5)**:** 613-627.

2. Chen S, Zambetti NA, Bindels EM, Kenswill K, Mylona AM, Adisty NM*, et al.* Massive parallel RNA sequencing of highly purified mesenchymal elements in low-risk MDS reveals tissue-context-dependent activation of inflammatory programs. *Leukemia* 2016 Sep; **30**(9)**:** 1938-1942.

3. Doulatov S, Notta F, Eppert K, Nguyen LT, Ohashi PS, Dick JE. Revised map of the human progenitor hierarchy shows the origin of macrophages and dendritic cells in early lymphoid development. *Nat Immunol* 2010 Jul; **11**(7)**:** 585-593.

4. Groschel S, Sanders MA, Hoogenboezem R, de Wit E, Bouwman BA, Erpelinck C*, et al.* A single oncogenic enhancer rearrangement causes concomitant EVI1 and GATA2 deregulation in leukemia. *Cell* 2014 Apr 10; **157**(2)**:** 369-381.

5. Subramanian A, Tamayo P, Mootha VK, Mukherjee S, Ebert BL, Gillette MA*, et al.* Gene set enrichment analysis: a knowledge-based approach for interpreting genome-wide expression profiles. *Proc Natl Acad Sci U S A* 2005 Oct 25; **102**(43)**:** 15545-15550.

6. Medyouf H, Mossner M, Jann JC, Nolte F, Raffel S, Herrmann C*, et al.* Myelodysplastic cells in patients reprogram mesenchymal stromal cells to establish a transplantable stem cell niche disease unit. *Cell Stem Cell* 2014 Jun 5; **14**(6)**:** 824-837.

7. van Dieren JM, Simons-Oosterhuis Y, Raatgeep HC, Lindenbergh-Kortleve DJ, Lambers MEH, van der Woude CJ*, et al.* Anti-inflammatory actions of phosphatidylinositol. *Eur J Immunol* 2011 Apr; **41**(4)**:** 1047-1057.

8. Mercurio F, Zhu H, Murray BW, Shevchenko A, Bennett BL, Li J*, et al.* IKK-1 and IKK-2: cytokine-activated IkappaB kinases essential for NF-kappaB activation. *Science* 1997 Oct 31; **278**(5339)**:** 860-866.

9. Bosman MC, Schepers H, Jaques J, Brouwers-Vos AZ, Quax WJ, Schuringa JJ*, et al.* The TAK1-NF-kappaB axis as therapeutic target for AML. *Blood* 2014 Nov 13; **124**(20)**:** 3130-3140.
